# Corneal and Coronary Calcification in Maintenance Hemodialysis: The Face Is No Index to the Heart

**DOI:** 10.1002/jbm4.10823

**Published:** 2023-12-13

**Authors:** Maria Beatriz C. N. Pessoa, Ruth Miyuki Santo, Aline A. de Deus, Eduardo J Duque, Shirley F. Crispilho, Vanda Jorgetti, Maria Aparecida Dalboni, Carlos Eduardo Rochitte, Rosa M. A. Moyses, Rosilene M. Elias

**Affiliations:** ^1^ Department of Post Graduation Universidade Nove de Julho (UNINOVE) Sao Paulo Brazil; ^2^ Department of Ophtalmology Universidade de Sao Paulo, Hospital das Clinicas HCFMUSP Sao Paulo Brazil; ^3^ Department of Internal Medicine Service of Nephrology, Universidade de Sao Paulo, Hospital das Clinicas HCFMUSP Sao Paulo Brazil; ^4^ Department of Radiology Universidade de Sao Paulo, Hospital das Clinicas HCFMUSP Sao Paulo Brazil

**Keywords:** calcification, coronary

## Abstract

Although the eyes are the main site of metastatic calcification in patients with chronic kidney disease (CKD), corneal and conjunctival calcification (CCC) is poorly evaluated in this population. Whether CCC correlates with coronary artery calcification remains unknown since studies so far have relied on methods with low sensitivity. Our objective was to test the relationship between CCC and coronary calcification based on tomography. This was a cross‐sectional study that included patients on maintenance dialysis. Clinical, demographic, and biochemical data (calcium, phosphorus, parathormone, alkaline phosphatase, and 25(OH)‐vitamin D) were recorded. Hyperparathyroidism was defined as parathyroid hormone (PTH) > 300 pg/mL. CCC was evaluated by anterior segment optical coherence tomography (AS‐OCT), and coronary calcium scores (Agatston method) were assessed by computed tomography. We compared no/mild with moderate/severe CCC. Twenty‐nine patients were included (49.6 ± 15.0 years, 62.1% female, on hemodialysis for 5.7 [2.7–9.4] years, 17.2% with diabetes mellitus, 75.9% with hyperparathyroidism). CCC was found in 82.7% of patients, with median scores of 9 (3, 14.5), ranging from 0 to 16. CCC was classified as absent/mild, moderate, and severe in 27.6%, 20.7%, and 51.7%, respectively. Coronary calcification was found in 44.8% of patients, with median scores of 11 (0, 464), varying from 0 and 6456. We found no significant correlation between coronary calcium scores and CCC (*r* = 0.203, *p* = 0.282). Hyperphosphatemia was more frequent in patients with moderate/severe CCC than in those with absent/mild CCC. We concluded that CCC was frequent in patients with CKD on dialysis and did not correlate with coronary calcium scores. Hyperphosphatemia appears to contribute to CCC. © 2023 The Authors. *JBMR Plus* published by Wiley Periodicals LLC on behalf of American Society for Bone and Mineral Research.

## Graphical abstract

Corneal calcification does not correlate with coronary artery calcification.

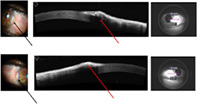

## Introduction

Metastatic calcification commonly occurs in the eyes of patients with chronic kidney disease (CKD). Corneal and conjunctival calcification (CCC) has long been recognized as the main site of extravascular calcification in patients with CKD on dialysis.^(^ ^1^, ^2^ ^)^ Arterial calcification contributes to increased mortality in this population.^(^ ^3^ ^)^

Moderate to severe calcification manifests in the aorta, peripheral vessels, and coronary and cardiac valves. Aortic calcification has an earlier onset and a higher prevalence than coronary artery calcification, although both are associated with cardiovascular outcomes. The pathogenesis of vascular calcification is complex and may be attributed to reactive oxidation species and bone and mineral disorders in the context of CKD (CKD‐MBD). Among the CKD‐MBD factors, calcium‐phosphate homeostasis has been implicated in the pathogenesis of vascular calcification in these patients.

Some authors have demonstrated the association between CCC and vascular calcification.^(^ ^1^, ^4^, ^5^, ^6^ ^)^ However, vascular calcification has been assessed using a variety of methodologies, including plain chest X‐ray^(^ ^1^ ^)^ or X‐ray of the pelvis and hands.^(^ ^4^ ^)^ In addition, the vascular site examined differs among studies.

The question remains as to whether there is a relationship between coronary artery calcification and CCC using a more reliable methodology that does not depend on the observer. We hypothesized that CCC and coronary calcification would be highly associated, using coronary tomography and anterior segment optical coherence tomography (AS‐OCT), methods with high sensitivity.

## Materials and Methods

This is a cross‐sectional study that included adult hemodialysis patients, recruited at the Nephrology Service of Hospital das Clinicas, Universidade de Sao Paulo, Brazil, during the period between August 2018 and November 2020. Inclusion criteria were >18 years old, stable on hemodialysis for at least 3 months, who agreed to participate and signed the written consent form. Exclusion criteria were ocular inflammation, previous ocular trauma, intravitreal silicon use, corneal transplantation, and chemical ocular burn.

Demographic, clinical, and laboratory data were obtained from charts and clinical interviews at inclusion. Written informed consent was obtained from each patient. The study protocol was approved by the institution's ethics committee on research on humans from the Universidade Nove de Julho (No. 3.05.3965) and the Hospital das Clinicas (No. 3.262.722).

The AS‐OCT was performed using a Cirrus HD 5000 OCT with an accessory corneal lens attached to the device's eyepiece, allowing visualization of the anterior segment of the eye and documentation of CCC. Images with a posterior acoustic shadow were considered calcium deposits. Since there was no existing definition, we propose the following classification of CCC: grade 0, without CCC; grade 1, isolated deposits in the conjunctiva only; grade 2, increased deposits in the conjunctiva as a line; grade 3, large deposits in the conjunctiva clumped together, nodule formation; and grade 4, corneal involvement (Supplementary Fig. S1). Drawing an imaginary line on the central meridian of both eyes, two quadrants, nasal and temporal, were obtained. The sum of scores in these regions was used to categorize CCC as absent/mild, moderate, or severe according to scores 0–4, 5–8, or 9–16, respectively.

Computed tomography (CT) scan was used to evaluate calcium deposits in the coronary arteries (Aquilion 64 TM – Toshiba TM Medical Systems Corporation, Otawara, Japan). Coronary calcification score was obtained according to the Agatston method using Vitrea TM 2 software (version 3.5; Vital Images Inc. Plymouth, MN, USA). Coronary calcification (in Hounsfield units [HU]) and area of all calcified lesions in the coronary arteries were categorized into very low risk (0), mild risk (1–99), moderate risk (100–299), and severe risk (≥300).^(^ ^7^ ^)^

Data are expressed as median (25, 75) or mean ± SD, as appropriate. We compared subjects with no/mild CCC and those with moderate/severe CCC using Student's *t* test or Mann–Whitney U test for continuous variables or chi‐squared or Fisher's exact test for nominal variables. Spearman's correlation coefficient was applied to test the relationship between independent variables. A *p*‐value <0.05 was considered statistically significant. The analyses were performed using SPSS version 26.0 (SPSS Inc., Chicago, IL, USA).

## Results

Of the 34 patients initially screened, five declined to participate and 29 were included (Table 1). The patients were relatively young, mostly female, and on dialysis for a median time of 5.7 years.

**Table 1:** Features of Patients According to Degree of Conjunctival and Corneal and Conjunctival Calcification (CCC) Evaluated Using Anterior Segment Optical Coherence Tomography (AS‐OCT)

Variable	All, *N* = 29	Absent/mild CCC, *N* = 14	Moderate/severe CCC, *N* = 15	*p*
Age, years	49.6 ± 15.0	48.8 ± 15.1	50.6 ± 15.7	0.755
Male sex, no. (%)	37.9	42.9	33.3	0.597
Dialysis duration, years	5.7 (2.2, 9.4)	5.5 (1.2, 9.7)	5.3 (4.1, 7.1)	0.747
Etiology of CKD, no. (%)				
Diabetes mellitus	5 (17.2)	2 (14.3)	3 (20.0)	0.348
Hypertension	8 (27.6)	6 (42.9)	2 (13.3)	
Glomerulopathy	6 (20.7)	2 (14.3)	4 (26.7)	
Other/unknown	10 (34.5)	4 (28.6)	6 (40.0)	
Total calcium, mg/dL	9.4 ± 1.0	9.3 ± 1.2	9.6 ± 0.8	0.419
Hypercalcemia, no. (%)	7 (24.1)	4 (28.6)	3 (20.0)	0.590
Ionized Calcium, mg/dL	4.96 ± 0.42	4.88 ± 0.35	5.05 ± 0.47	0.288
Phosphate, mg/dL	5.2 ± 1.9	4.5 ± 1.6	5.7 ± 2.3	0.069
Hyperphosphatemia, no. (%)	11 (37.9)	3 (21.4)	8 (53.3)	0.039
Calcium*phosphate product	49.2 ± 20.4	41.9 ± 14.2	55.9 ± 23.4	0.063
AP, U/L	140 (94, 335)	122 (90, 400)	146 (95, 316)	0.533
PTH, pg/mL	684 (257, 1309)	546 (216, 1366)	725 (437, 1293)	0.477
PTH >300 pg/mL, No (%)	22 (75.9)	9 (64.3)	13 (86.7)	0.159
25(OH)‐vit.D, ng/mL	34.2 ± 14.2	34.9 ± 16.6	34.2 ± 12.3	0.903
Cinacalcet, mg/day	30 (0, 37.5)	0 (0–37.5)	30 (0.30)	0.533
Calcitriol, mg/week	30 (0, 30)	0 (0, 37.5)	30 (0, 30)	0.566
Paricalcitol, mg/week	3.0 (0, 9.0)	2.0 (0, 9.0)	6.0 (1.0, 9.0)	0.466
Calcium carbonate, g/day	0 (0, 0.5)	0 (0, 0.75)	0 (0–0.62)	0.769
Sevelamer g/day	3.5 (0, 9.0)	5.5 (0–9)	1.6 (0–7.2)	0.477
Cholecalciferol*1000 UI/week	6.2 (0, 22.5)	5.0 (0, 15.6)	9.4 (0–25.0)	0.635
Coronary calcification				
Agatston scores, HU	11 (0, 464)	0 (0, 88.2)	21.6 (0, 865)	0.376
Classification, %				
0 HU	48.3	57.1	40.0	0.673
1–99 HU	20.7	21.4	20.0	
100–299 HU	6.9	7.2	6.7	
≥300 HU	24.1	14.3	33.3	

*Note*: Values are expressed as mean ± SD or median (25, 75%), unless otherwise specified.

Abbreviations: AP, alkaline phosphatase; CKD, chronic kidney disease; HU, Hounsfield units; PTH, parathyroid hormone; 25 (OH)Vit.D, 25 (OH) vitamin D.

**Fig. 1:**
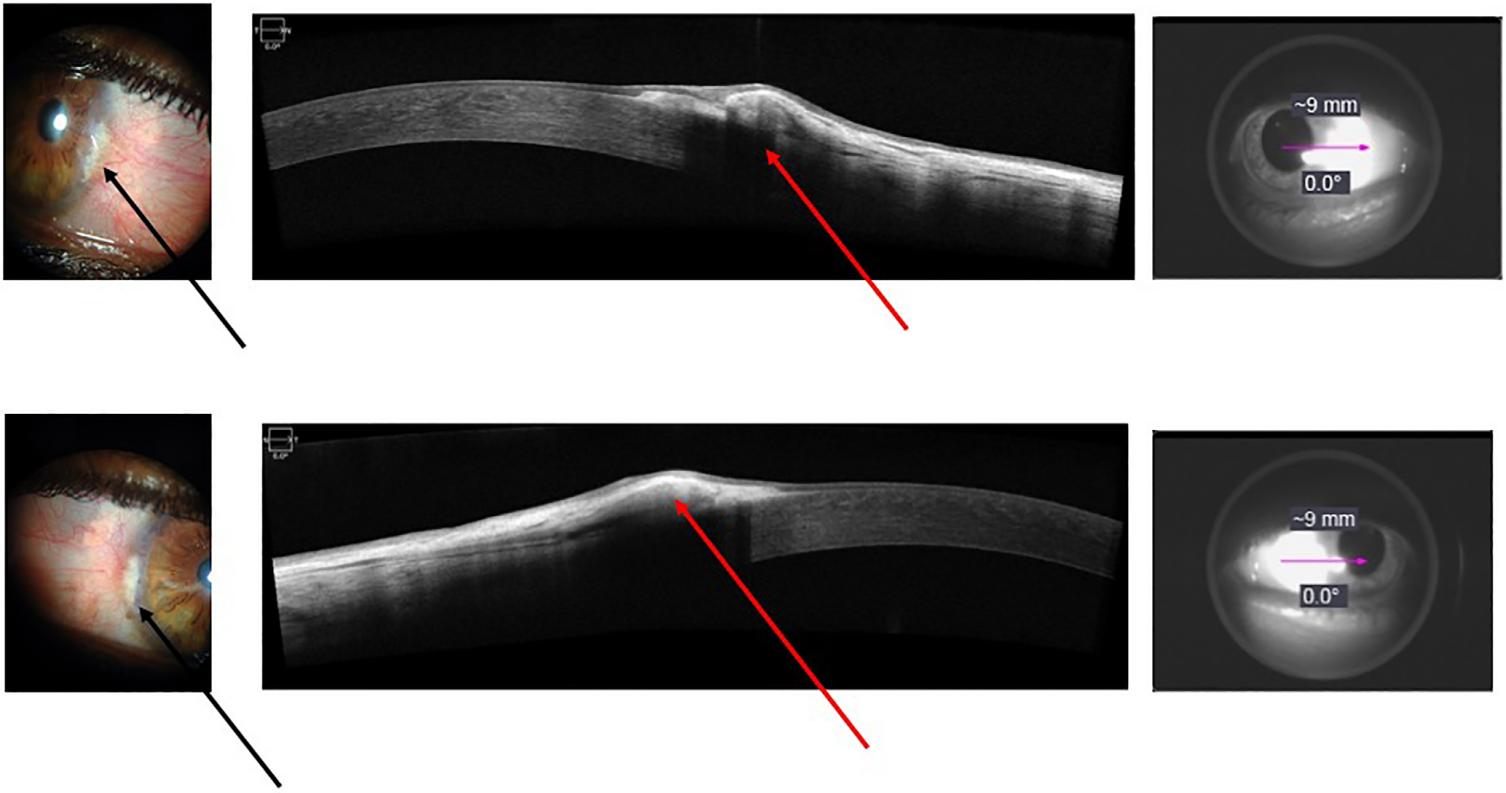
Images obtained from a patient with corneal and coronary calcification (CCC) visualized by slit lamp and confirmed with anterior segment optical coherence tomography (AS‐OCT). Left to right: slit‐lamp photographs of right eye CCC at temporal and nasal angles (upper and lower panels, respectively) showing a calcified area (black arrows); middle: image from AS‐OCT showing posterior acoustic shadow and elevation of corneal surface (red arrows), considered calcium deposits, followed by a guide image from where photo was taken.

CCC was observed in 24 patients (82.7%). Images obtained from a given patient are illustrated in Fig. 1. CCC scores correlated with phosphate levels (*r* = 0.406, *p* = 0.029) and calcium*phosphate product (*r* = 0.415, *p* = 0.025). Absent/mild CCC was found in 48.3% and moderate/severe CCC in 51.7%. Hyperphosphatemia was more frequent in the last group (*p* = 0.039). There was no other clinical or laboratory difference between groups.

Coronary calcification was found in 44.8% of patients, with median scores of 11 (0, 464), varying from 0 to 6456. Coronary calcium scores correlated with age (*r* = 0.621, *p* = 0.0001).

There was no significant correlation between coronary calcium scores and CCC (*r* = 0.203, *p* = 0.282). The severity of coronary calcification was similar among patients with absent/mild CCC and those with moderate/severe CCC (Table 1).

## Discussion

This pilot study provides novel information on the association between the eye and coronary calcification. First, we found no relationship between coronary calcium scores and CCC scores. It should be noted that, although we have evaluated coronary calcium, the aorta is the most prominent site of calcification in patients with CKD.^(^ ^8^ ^)^ Second, we describe, for the first time, a classification of CCC based on AS‐OCT, which is more sensitive than the clinical examination to evaluate calcification.

CCC was found in 82.7% of patients, in agreement with previous data.^(^ ^2^, ^5^, ^9^ ^)^ Using CT imaging, previous studies found a two‐ to fivefold increase in coronary artery calcification in patients on dialysis.^(^ ^10^ ^)^ We confirmed the high prevalence of coronary calcium calcification in our study population.

Vascular calcification occurs when there is a disruption in the balance between inhibitors of calcification and active inducers.^(^ ^11^ ^)^ Phosphate is the main identified player in explaining vascular calcification.^(^ ^12^ ^)^ Interestingly, there is a description of corneal calcification after irrigation of phosphate‐buffered saline after burns,^(^ ^13^ ^)^ the use of artificial tears,^(^ ^14^ ^)^ and a study of an ex vivo eye irritation test showing calcification following phosphate eye drop.^(^ ^13^ ^)^ Phosphate and the calcium phosphate product have been implicated in the symptomatology of red and dry eyes in patients with CKD.^(^ ^15^ ^)^ Of note, most of our patients have secondary hyperparathyroidism, a condition associated to loss of calcification inhibitors such as fetuin, inorganic pyrophosphate, and matrix Gla protein, causing vascular calcification.

We did not confirm previous studies that demonstrated an association between CCC and vascular calcification.^(^ ^1^, ^4^, ^5^, ^6^ ^)^ At least two reasons might explain the incongruence between the findings of this study and those of others. First, whereas we used AS‐OCT to evaluate CCC, previous researchers used a slit lamp. Second, mostprevious studies assessed aortic and heart valve calcification using X‐ray or ultrasound, whereas we evaluated coronary artery calcification using tomography.

The results of our study must be interpreted considering some limitations, such as the small sample size and the high prevalence of secondary hyperparathyroidism, which precludes generalizing the results. The strengths of our study consist in its use of a more specific and sensitive methodology, including coronary artery tomography and AS‐OCT. Furthermore, we described for the first time a proposal for a CCC classification based on AS‐OCT.

In summary, we found no obvious association between CCC and coronary artery calcification. However, further investigation with a larger sample size is needed to confirm this finding. Our result suggests a different physiopathology of both calcification sites, coronary and eyes. In this way, we previously demonstrated an improvement in CCC after parathyroidectomy,^(^ ^16^ ^)^ whereas coronary artery calcification seems to be a more complicated site to recover from calcification.^(^ ^17^ ^)^ Hyperphosphatemia was identified as a risk factor for corneal and conjunctival calcification. CKD‐MBD is a recognized risk factor for calcification. In this regard, reduction of calcium load, avoiding oral calcium carbonate, and reducing the dialysate calcium concentration may attenuate progression. An ophthalmological examination is strongly recommended in patients with CKD since it is an easy method of recognizing calcification in these patients.

## Funding Information

The authors Rosa M. A. Moyses and Rosilene M. Elias disclosed receipt of financial support from the Conselho Nacional de Desenvolvimento Científico e Tecnológico – CNPQ (grants 303545/2020‐8 and 304901/2021‐0, respectively). These financial supports had no role in study design; collection, analysis, and interpretation of data; writing of the report; or the decision to submit the report for publication.

## Conflict of Interest

The authors declare that they have no conflicts of interest to declare that could appear to influence the work reported in this study.

## Author Contributions

**Maria Beatriz C. N. Pessoa:** Conceptualization; data curation; methodology; writing – review and editing. **Ruth Miyuki Santo:** Conceptualization; data curation; investigation; methodology; project administration; supervision; visualization; writing – original draft; writing – review and editing. **Aline A. de Deus:** Investigation; writing – review and editing. **Eduardo J Duque:** Investigation; writing – review and editing. **Shirley F. Crispilho:** Investigation; writing – review and editing. **Vanda Jorgetti:** Data curation; project administration; visualization; writing – review and editing. **Maria Aparecida Dalboni:** Visualization; writing – review and editing. **Carlos Eduardo Rochitte:** Methodology; resources; writing – review and editing. **Rosa MA Moyses:** Data curation; formal analysis; visualization; writing – review and editing. **Rosilene M. Elias:** Conceptualization; data curation; formal analysis; investigation; project administration; supervision; writing – original draft; writing – review and editing.

### Peer Review

The peer review history for this article is available at https://www.webofscience.com/api/gateway/wos/peer‐review/10.1002/jbm4.10823.

## Supporting information


**Supplementary Fig. S1.** Illustrative CCC images captured by anterior segment optical coherence tomography (AS‐OCT). Drawing an imaginary line on the central meridian of both eyes, two quadrants, nasal and temporal, were obtained. The sum of scores in these regions was used to categorize the CCC as mild, moderate, or severe according to scores 0–4, 5–8, or 9–16, respectively. (A–D) Illustrative images of CCC grade 1 (isolated deposits in the conjunctiva only), 2 (increased deposits on the conjunctiva as a line), 3 (large deposits on the conjunctiva clumped together, nodule formation), and 4 (corneal involvement).
