# Supplementary figures and images for: Corneal and Coronary Calcification in Maintenance Hemodialysis: The Face Is No Index to the Heart
Source: JBMR Plus. 2023 Dec 13;7(12):e10823. doi: 10.1002/jbm4.10823 (PMC10731104; doi:10.1002/jbm4.10823)

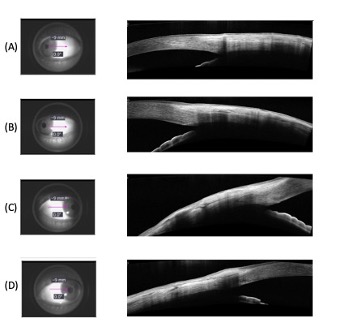

Supplement: Supplementary file 1 — Supplementary Fig. S1. Illustrative CCC images captured by anterior segment optical coherence tomography (AS‐OCT). Drawing an imaginary line on the central meridian of both eyes, two quadrants, nasal and temporal, were obtained. The sum of scores in these regions was used to categorize the CCC as mild, moderate, or severe according to scores 0–4, 5–8, or 9–16, respectively. (A–D) Illustrative images of CCC grade 1 (isolated deposits in the conjunctiva only), 2 (increased deposits on the conjunctiva as a line), 3 (large deposits on the conjunctiva clumped together, nodule formation), and 4 (corneal involvement). [file JBM4-7-e10823-s001.jpg]
